# Cathepsin B Localizes in the Caveolae and Participates in the Proteolytic Cascade in Trabecular Meshwork Cells. Potential New Drug Target for the Treatment of Glaucoma

**DOI:** 10.3390/jcm10010078

**Published:** 2020-12-28

**Authors:** April Nettesheim, Myoung Sup Shim, Angela Dixon, Urmimala Raychaudhuri, Haiyan Gong, Paloma B. Liton

**Affiliations:** 1 Department of Ophthalmology & Pathology, Duke University, Durham, NC 27705, USA; april.nettesheim@duke.edu (A.N.); myoungsup.sim@duke.edu (M.S.S.); angela.norman@duke.edu (A.D.); urmimala.raychaudhuri@duke.edu (U.R.); 2 Department of Ophthalmology, Boston University School of Medicine, Boston, MA 02118, USA; hgong@bu.edu

**Keywords:** glaucoma, trabecular meshwork, cathepsin B, fibrosis, TGFβ, ECM, PAI-1, proteolytic cascade

## Abstract

Extracellular matrix (ECM) deposition in the trabecular meshwork (TM) is one of the hallmarks of glaucoma, a group of human diseases and leading cause of permanent blindness. The molecular mechanisms underlying ECM deposition in the glaucomatous TM are not known, but it is presumed to be a consequence of excessive synthesis of ECM components, decreased proteolytic degradation, or both. Targeting ECM deposition might represent a therapeutic approach to restore outflow facility in glaucoma. Previous work conducted in our laboratory identified the lysosomal enzyme cathepsin B (CTSB) to be expressed on the cellular surface and to be secreted into the culture media in trabecular meshwork (TM) cells. Here, we further investigated the role of CTSB on ECM remodeling and outflow physiology in vitro and in CSTB^ko^ mice. Our results indicate that CTSB localizes in the caveolae and participates in the pericellular degradation of ECM in TM cells. We also report here a novel role of CTSB in regulating the expression of PAI-1 and TGFβ/Smad signaling in TM cells vitro and in vivo in CTSB^ko^ mice. We propose enhancing CTSB activity as a novel therapeutic target to attenuate fibrosis and ECM deposition in the glaucomatous outflow pathway.

## 1. Introduction

The trabecular meshwork (TM) is a tissue located in the anterior segment of the eye, which has a major role in regulating intraocular pressure (IOP), by controlling the resistance to aqueous humor (AH) outflow from the eye. Functional failure of the TM causes ocular hypertension and predisposes the development of primary open angle glaucoma (POAG), a group of eye diseases leading cause of permanent blindness worldwide [1,2].

The TM consists of sheets of connective tissue beams through which AH must flow before exiting the eye. Each beam is composed of a central elastic core surrounded by collagen fibers embedded in a ground substance, lined by TM endothelial cells. With aging and in disease, the trabecular beams become thicker due to thickening of the basement membrane underlying the trabecular cells or changes in the extracellular matrix (ECM) within the central core. In the TM of glaucomatous eyes, deposition of long-spacing collagen is also frequently observed [3]. The exact causes responsible for this thickening of the beams and deposit of ECM remain unknown, but are likely due to excessive ECM synthesis, decreased proteolytic degradation, or both [4]. Excessive synthesis possibly plays a role because the aqueous humor (AH) of patients with POAG contains elevated concentrations of TGF-β2 [5,6], a cytokine that promotes ECM deposition and increases outflow resistance [6,7]. Degradation is controlled extracellularly by the action of proteases. Regulation of the ECM composition in the TM is known to play a major role in both outflow pathway physiology and pathophysiology [4,8,9]; however, the exact mechanisms underlying ECM remodeling with IOP changes and those associated with its alteration in glaucoma remain poorly understood [10].

Despite the general belief that matrix metalloproteinases (MMPs) are the only group of enzymes responsible for ECM degradation, emerging evidence in other tissues suggests that other proteases or the coordinated action of several types of proteases, other than just MMPs, participate in bulk matrix degradation and ECM remodeling [11]. In this line, previous studies conducted in our laboratory reported for the first time a role of the lysosomal cysteine protease cathepsin B (CTSB) in intracellular degradation of ECM components in TM cells [12]. We found partially degraded ECM products associated with active CTSB in lysosomal compartments. Moreover, CTSB was found to be constitutively expressed in the cell surface and secreted to the culture media in its inactive pro-form. CTSB was also detected in AH samples. Surface expression and secretion of cathepsins are not unique to TM cells. Although preferentially located in lysosomes, specific subtypes of cathepsins (CTSB, CTSL, CTSK, CTSD) have been located on the cell surface as well as secreted into the extracellular space in different cell types, preferentially of mesenchymal origin, either constitutively or under stress conditions [13,14,15]. In particular, surface CTSB has been localized in caveolae in HUVEC endothelial and colorectal carcinoma cells [16,17]. Caveolae are lipid-rich invaginations of the plasma that play a key role in regulating cell signaling, lipid homeostasis, and adaptation to membrane tension. Emerging studies are now showing that caveolae are also important regulators of ECM remodeling, acting as focal proteolytic degradation sites. CTSB activity at the cellular membrane has been associated with the pericellular degradation of ECM and basement membrane proteins and contribute to cell migration in cancer cells [15,17,18]. Interestingly, CTSB has been also shown to promote epithelial–mesenchymal transition (EMT) in tumor cells and contribute to tissue fibrosis [19].

Given the relevance of fibrosis and EMT transition in outflow pathway pathophysiology, we decided to follow up on our initial observations and to further investigate a role of CTSB in ECM remodeling and TM tissue function. Here, we show for the first time that surface CTSB localizes in the caveolae and participates in the pericellular proteolytic cascade in TM cells and TGFβ signaling.

## 2. Experimental Section

### 2.1. Cell Culture

Primary human TM cells were isolated from dissected TM tissue from discarded corneal rims after surgical corneal transplantation at Duke University Eye Center and maintained as described earlier [20]. Cells were passaged when confluent at a 1:2 ratio. Cells from passage 4–8 were used in this study. HTM cells were characterized by morphology and by the upregulation of myocilin in response to dexamethasone treatment, in accordance to the consensus recommendations for TM cell isolation, characterization, and culture [21]. The protocols involving the use of human tissue were consistent with the tenets of the Declaration of Helsinki.

### 2.2. Preparation of Caveolae-Enriched Fractions

Caveolae-enriched fractions were prepared using the Caveolae Isolation Kit (Sigma-Aldrich, St. Louis, MO, USA) following the manufacturer’s instruction. This kit is based on the resistance of caveolae to solubilization in ice cold Triton X-100. Briefly, one confluent 100 mm-tissue culture dish of hTM cells was washed twice with cold 1 × PBS and lysed on ice in proprietary Lysis Buffer containing 1% Triton X-100. Fractions were fractionated by OptiPrep density gradient ultracentrifugation and collected in 1 mL fractions. Caveolin-1 (Cav1) was used as a marker for caveolae-enriched fractions (fractions 2–5).

### 2.3. siRNA Transfection

Human primary TM cells were transfected with siCTSB (25 pmol, Santa Cruz Biotechnology, Dallas, TX, USA; sc-29238) using Lipofectamine RNAiMAX protocol (Invitrogen) following the manufacturer’s instructions. A non-targeting siRNA (siNC) was used as control.

### 2.4. Whole-Cell and Tissue Lysates Preparation

Cells and dissected iridocorneal region tissue were washed twice in PBS and lysed for 30 min in RIPA buffer (Millipore Sigma, Burlington, MA, USA; R0278) containing 1:100 Halt protease and phosphatase inhibitor cocktail (Thermo Scientific, Waltham, MA, USA; 78842). After sonication for one minute on ice, lysates were clarified by centrifugation. Protein concentration was determined using a protein assay kit (Micro BCA, Thermo Scientific, Waltham, MA, USA; 23235).

### 2.5. Western Blot Analysis

For WB analysis, proteins (2.5–10 μg), cell culture medium (3.5–20 μL), or caveola-enriched fractions (15 μL) were separated by SDS-PAGE (10–15%) and transferred to PVDF membrane (Bio-Rad). Membranes were blocked with 5% non-fat dry milk in PBS-T (0.1% Tween-20) for 1 h at room temperature or overnight at 4 °C and incubated with primary antibodies diluted in blocking buffer overnight at 4 °C. Membranes were washed three times with PBS-T and incubated with peroxidase-conjugated secondary antibodies for 1–2 h at room temperature. Bands were detected using chemiluminescence substrate (HyGlo Quick Spray, Denville, Swedesboro, NJ, USA; E2400). Blots were scanned using ChemiDoc Image System and analyzed by densitometry using Image J. ACTB or GAPDH were used for normalization. Primary antibodies and dilutions used in the study are listed in Table 1.

### 2.6. RNA Isolation and Quantitative Real-Time PCR

Total RNA was isolated from human TM cells using RNeasy kit (Qiagen) according to the manufacturer’s protocol and treated in-column with DNase I (Qiagen). RNA yields were measured using Nanodrop spectrophotometry (NanoDrop 1000, Thermo Scientific, Waltham, MA, USA). First-strand cDNA was synthesized from total RNA (1 μg) by reverse transcription using oligo(dT) primer and reverse transcriptase (SuperScript First Strand System, Thermo Scientific, Waltham, MA, USA). Real-time PCR was performed in a 20 μL mixture containing 1 μL of the cDNA preparation diluted five times, 10 μL master mix (SsoFast EvaGreen Supermix, Bio-Rad, Hercules, CA, USA), and 500 nm of each primer in a thermocycler system (iCycler iQ; Bio-Rad, Hercules, CA, USA) using the following PCR parameters: 95 °C for 5 min, followed by 50 cycles of 95 °C for 15 s, 60 °C for 15 s, and 72 °C for 15 s. The fluorescence threshold value (Ct) was calculated using the thermocycler system software. The absence of nonspecific products was confirmed by both the analysis of the melt curves and by electrophoresis in 3% acryl-agarose gels. β-Actin and GAPDH served as an internal standard of mRNA expression. The change (x-fold) was calculated with the formula 2^−ΔΔCt^, where ΔCt = Ct_gene_ − Ct_Act_, and ΔΔCt = ΔCt_Exp_ − ΔCt_Con_. The sequences of the primers used for the amplifications are: PAI-1: TGF TGG TGA ATG CCC TCT ACT and CGG TCA TTC CCA GGT TCT CTA.

### 2.7. In Situ Zymography

Live cell proteolysis was assayed as previously described [12], with some modifications. Briefly, 24-well plates were coated with 50 μL of 1.5% (*w*/*v*) gelatin (Sigma, G1393) containing 25 μg/mL DQ-gelatin or DQ-Collagen I (Thermo Fisher Scientific, Waltham, MA, USA; D12054) and incubated on ice for 15 min to solidify. Cells (2.5 × 10^4^) were seeded onto coated wells and incubated at 5% CO_2_, 37 °C, for 48 h. CA074Me (40 µM; Enzo life science, Farmingdale, NY, USA; BML- PI 126), E-64d (10 µM; Sigma-Aldrich, St. Louis, MO, USA; E8640) or marimastat (10 μM; Sigma-Aldrich, St. Louis, MO, USA; M2699) were added to the culture media to inhibit CTSB, cysteine cathepsins, or MMP activities, respectively. Fluorescence peptides released by the enzymatic cleavage of the substrate at the pericellular region was observed in live cells using the CELENA^®^ X High Content Imaging System (Logos biosystems, Annandale, VA, USA) coupled with a stage top incubation system (Ibidi USA, Inc., Fitchburg, WI, USA). Nuclei were stained by 15 min incubation with Hoechst 33342. Intracellular proteolysis of internalized DQ-substrates was quantified by flow cytometry in FL1 channel.

### 2.8. Mechanical Stretch

Cyclic mechanical stretch (CMS, 8% elongation, 1Hz) was applied using the Flexcell^®^ FX-5000™ Tension System (Flexcell International Corp, Burlington, NC, USA), as described previously [22].

### 2.9. ELISA

Secreted TGFβ1 and TGFβ2 were measured by ELISA (Invitrogen, BMS249-4 and BMS254, respectively) in 100 μL of clarified culture media, according to the manufacturer’s protocol.

### 2.10. CTSB^ko^ Mice

CTSB^ko^ mice were obtained from Dr. Henriette Van Praag at Florida Atlantic University (Boca Raton, FL, USA) [23]. Generation of these mice has been described in [24]. Mice were maintained and aged in situ. Genotyping was performed by PCR in digested tail genomic DNA using the following primers with a temperature annealing of 57.5 °C for 45 s (F: GGT TGC GTT CGC TGA GG; R: AAC AAG AGC CGC AGG AGC). CTSB^ko^ and littermates CTSB wild-type (males and females) were used for experimental purposes. Animals were maintained under a 12 h light/dark cycle, fed a standard mouse diet, and provided with water ad libitum. Animal euthanasia was performed via CO_2_ asphyxiation, followed by bilateral thoracotomy, prior to immediate enucleation. Enucleated eyes destined for electron microscopy (EM) were fixed under perfusion in 2% glutaraldehyde/0.1 M cacodylate buffer. Tissues destined for WB analysis were immediately dissected and flash frozen on dry ice. All procedures were reviewed and approved by the Institutional Animal Care and Use Committee of Duke University and were performed in accordance with the ARVO Statement for the Use of Animals in Ophthalmic and Vision Research and the National Institutes of Health Guide for the Care and Use of Laboratory Animals.

### 2.11. IOP Measurements

IOP was measured between 10 a.m. and 12 p.m., bi-monthly in isoflurane-anesthetized mice using a TonoLab rebound tonometer (Colonial Medical Supply, Franconia, NH, USA). Six measurements, taken within 5 min after anesthesia, were collected per eye and averaged to obtain a single IOP value per eye for each session.

### 2.12. Ex-Vivo Outflow Facility

Eyes were enucleated within 5 min of death and kept in a warmed solution of 5.5 mM glucose in Dulbecco’s PBS with calcium and magnesium (DBG) until perfusion (15–20 min). Outflow facility measurements were conducted ex-vivo using the iPerfusion MK V system [25]. For this, eye cups were affixed with a small amount of cyanoacrylate glue (Locktite, Henkel Corporation, Rocky Hill, CT, USA) to platforms within heated baths filled with DBG. XYZ micromanipulators (World Precision Instruments, Sarasota, FL, USA) were used to cannulate the anterior chamber of each eye with a glass microneedle. The glass microneedles were manufactured in-house by pulling glass capillary tubes (PN-3 Puller, Narishge Scientific Instrument Lab, Tokyo, Japan) and sharpened to a 20 degree bevel (BV-10, Sutter Instrument Company, Novato, CA, USA). An initial pressure of 8 mmHg for a period of 30 min was applied to allow acclimatization of the eyes to pressure and temperature environment. A nine-step post acclimatization protocol was conducted with applied pressures of 5.0, 6.5, 8.0, 9.5, 11.0, 12.5, 14.0, 15.5, and 17.0 mmHg before returning to 8.0 mmHg. Outflow facility was calculated with the iPerfusion integrated software [25]. 

### 2.13. Electron Microscopy (EM)

Enucleated eyes were post-fixed for 24 h in 2% glutaraldehyde/0.1 M cacodylate buffer solution and transferred to cold 3% glutaraldehyde/0.1 M cacodylate buffer for 2 h. Eyes were then washed in cacodylate buffer, post-fixed in 2% OsO4/0.1 M cacodylate, and dehydrated though increasing ethanol gradient ending in two cycles of propylene oxide. Tissue was infiltrated by immersion in a 1:1 mixture of propylene oxide and Epon 812 resin under a vacuum for 4–10 h. Labeled molds were then heated at 68 °F for 8–10 h. Sections were cut at 65 nm thick using a Leica EM CU7 and contrast stained with 2% uranyl acetate/4% lead citrate solution. Ultrathin sections were visualized on a JEM-1400 transmission electron microscope (JEOL) using an ORIUS (1000) CCD camera. Electron microscopic images of trabecular meshwork at lower magnification (X3000–4000), and higher magnification (X10000–12000) from four wide-type and three CTSB KO mouse eyes were analyzed and compared.

### 2.14. Statistical Analysis

All in vitro experimental procedures were repeated at least three times in independent experiments with hTM primary cultures from different donors. Animal studies were conducted with *n* = 4 to 11, as indicated for each particular experiment. Data were presented as the mean ± S.D. Statistical analysis was done in GraphPad Prism software using Student’s *t*-test or 2-way ANOVA with Tukey post-test for multiple comparisons. *p* < 0.05 was considered to be statistically significant.

## 3. Results

### 3.1. Cathepsin B Localizes in Caveolae with Components of the Proteolytic Cascade and Is Secreted into the Culture Media in Primary Cultures of TM Cells

**Figure 1:**
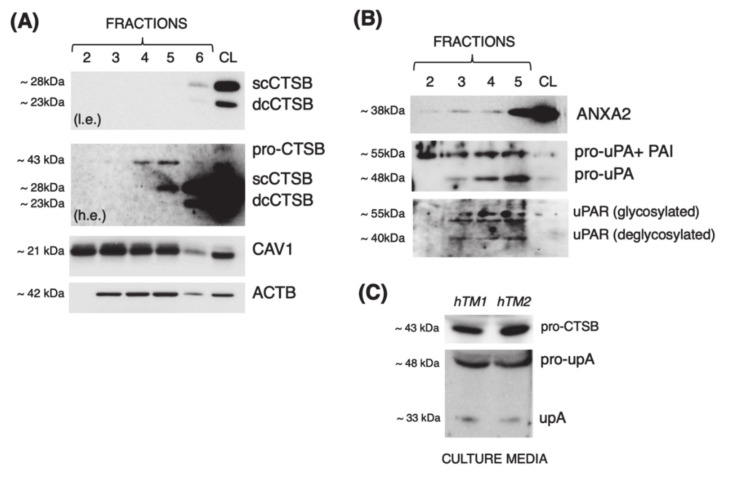
Localization of CTSB and members of the proteolytic cascade in the caveolae. Representative WB showing expression of (**A**) CTSB (pro-CTSB, sc-CTSB, dc-CTSB) and CAV1, or (**B**) ANXA2, pro-uPA and uPAR in caveolae-enriched fractions (2–5, 15 μL) and cell lysates (CL) from hTM cells. l.e.: lower exposure; h.e.: higher exposure. (**C**) Representative WB showing expression of pro-CTSB, pro-uPA, and uPA in conditioned media (15 μL) from hTM cells.

Our laboratory previously reported that CTSB is constitutively expressed at the cell surface in porcine TM cells and secreted into the culture media in its inactive pro-CTSB form [12]. We want to investigate whether membrane CTSB localizes to caveolae, as described in HUVEC endothelial and human colorectal carcinoma cells [16,17]. For this, we purified caveolae-enriched fractions from primary human TM cultures and evaluated CTSB expression in the collected protein fractions by WB. We first analyzed the expression of the caveolae marker, caveolin 1 (CAV1). As seen in Figure 1A, CAV1 was mostly found in the low-density fractions 2 through 5, hereafter designated as caveolae fractions, with a sharp decreased in high-density fraction 6. Excitingly, pro-CTSB and active single-chain CTSB (scCTSB) were detected in the caveolae-enriched fractions 4–5 and 5, respectively. It should be noted that double-chain CTSB (dcCTSB) was found in whole-cell lysates (CL) and fraction 6, but it was not localized in the caveolae-enriched fractions. scCTSB to dcCTSB transition occurs in the lysosomes; therefore, its expression is not anticipated in the caveolae. It should also be noted that the caveolae purification method is based on the unique resistance of caveolae to solubilization in ice-cold Triton X-100. While caveolae do not solubilize, endosomes and lysosomes (potential contaminants) do [26]. We additionally investigated the presence in the caveolae of components of the proteolytic cascade urokinase plasminogen activator (uPA), its receptor (uPAR), and that of annexin A2 (ANXA2), a proposed binding partner of CTSB and the cell membrane. As observed in Figure 1B, all of them (uPA, uPAR and ANXA2) were identified in the caveolae-enriched fractions. A band of ~55 kDa immunoreacting with anti-uPA antibody, referred in the literature as uPA bound to plasminogen activator inhibitor type-1 (PAI-1), its natural inhibitor, was also identified. We next confirmed whether, as we previously reported in porcine TM cells, CTSB was secreted by human TM cells. For this, we analyzed CTSB expression in conditioned media from cultured human TM cells; uPA was used as control. Similar to our previous findings, pro-CTSB, but not active CTSB, was detected (Figure 1C). Altogether, these data strongly indicate that CTSB localizes in the caveolae, together with components of the proteolytic cascade, and it is secreted into the culture media in primary cultures of human TM cells.

### 3.2. Cathepsin B Mediates Pericellular Proteolysis of ECM in TM Cells

**Figure 2:**
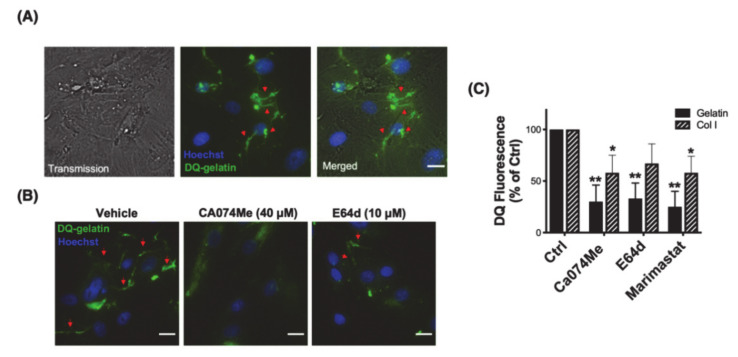
CTSB mediates pericellular proteolysis in hTM cells. (**A**) Representative image of pericellular proteolysis in hTM cells. Proteolytic degradation products of DQ-gelatin (green fluorescence) were monitored as described in materials and methods. (**B**) Representative image of pericellular proteolysis in hTM cells in the presence of the CTSB or cysteine cathepsin inhibitors Ca074Me and E64d, respectively. (**C**) Flow cytometry quantification of DQ-gelatin and DQ-Col I proteolytic degradation products in the presence and absence of Ca074Me, E64d, or marimastat (MMP inhibitor). Data are shown as the mean ± S.D. * *p* < 0.05; ** *p* < 0.01, *n* = 3. Hoechst (blue fluorescence) was used to stain nuclei. Red arrows represent the pericellular degradation of DQ-gelatin. Scale bars: 20 μm.

The finding that CTSB localizes in caveolae with other members of the proteolytic cascade opens the question of whether CTSB participates in the pericellular degradation of ECM at the cell surface. This was investigated by in situ live zymography using DQ-gelatin, a highly quenched fluorescein-labeled gelatin that becomes fluorescence upon proteolytic digestion. Human TM cells were grown onto gelatin containing DQ-gelatin (25 μg/mL). Proteolytic degradation of DQ-gelatin was monitored using the Celena X imaging system. Degradation products of DQ-gelatin were observed at the cellular periphery after 48 h of culture (Figure 2A, green fluorescence, red arrows). We next repeated the experiment in the presence of the CTSB or the cysteine protease inhibitors, Ca074Me (40 μM) and E64d (10 μM), respectively. As seen in Figure 2B, reduced gelatinolytic activity (decreased green fluorescence) was observed at the pericellular region in the presence of the inhibitors. We additionally quantified by flow cytometry the effect of Ca074Me, E64d, and marimastat, an MMP inhibitor, on the intracellular degradation of DQ-gelatin and DQ-collagen I (Figure 2C). Similar to our results in porcine TM cells [12], degradation of DQ-substrates was significantly blocked by inhibiting CTSB (gelatin: 30.05 ± 16%, *p* < 0.01, *n* = 3; Col I: 58 ± 17%, *p* < 0.01, *n* = 3) and cysteine cathepsin activities (gelatin: 33 ± 15%, *p* < 0.05, *n* = 3; Col I: 67 ± 19%, *n* = 3), at same levels of those observed with the MMP inhibitor (gelatin: 25 ± 15%, *p* < 0.01, *n* = 3; Col I: 58 ± 16%, *p* < 0.05, *n* = 3). Together, the results strongly confirm a role of CSTB and cysteine cathepsins in the pericellular and intracellular degradation of ECM components.

### 3.3. Inhibition of CTSB Upregulates and Increases the Secretion of Plasminogen Activator Inhibitor 1 (PAI-1)

**Figure 3:**
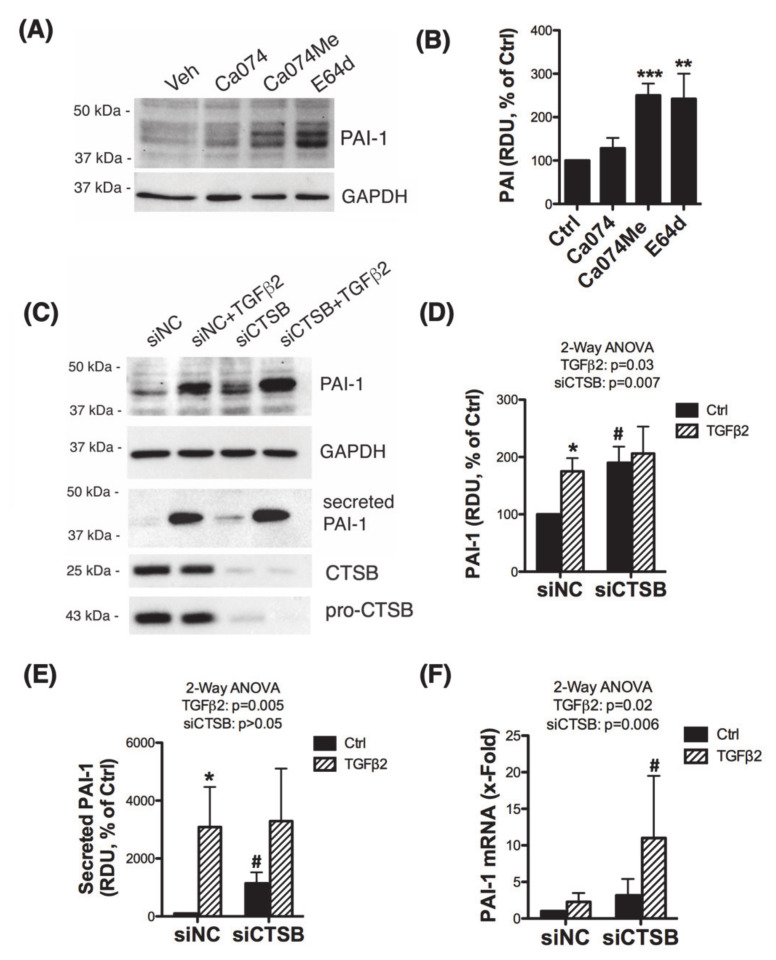
CTSB inhibition increases production and secretion of PAI-1. (**A**) Protein levels of PAI-1 evaluated by WB in whole cell lysates (5 μg) from primary hTM cells treated with Ca074 (40 μM), Ca074Me (40 μM), and E64d (10 μM) for 24 h. Relative PAI-1 protein levels calculated from densitometric analysis of the bands are represented in (**B**). Data are shown as the mean ± S.D. ** *p* < 0.01; *** *p* < 0.001, *t*-test, *n* = 3. (**C**) Protein levels of PAI-1 evaluated by WB in whole cell lysates (5 μg) and culture media (15 μL) from primary hTM cells transfected for 72 h with siCTSB followed by TGFβ2 treatment (10 ng/mL, 48 h). Relative PAI-1 protein levels calculated from densitometric analysis of the bands are represented in (**D**,**E**). Data are shown as the mean ± S.D. * *p* < 0.05 2-way ANOVA with Tukey comparison tests, *n* = 3. (**F**) mRNA expression levels of PAI-1 in siCTSB-transfected TGFβ2-treated hTM cells calculated by qPCR. Data are shown as the mean ± S.D. *p* < 0.05 2-way ANOVA with Tukey comparison tests, *n* = 3. * compares TGFβ2-treated to non-treated controls; ^#^ compares siCTSB to siNC.

Several studies have shown the ability of CTSB to degrade ECM either intracellularly, extracellularly, or both by initiating a proteolytic cascade that involves uPA, plasminogen (PLG), and PAI-1 [11,14,17,27,28,29,30,31]. We tested the effect of CTSB inhibition on the expression levels of the components of the proteolytic cascade PLG, uPA, and PAI-1 by WB. For this, we treated three independent primary cultures of human TM cells with Ca074, Ca074Me, or E64d for 24 h. Intriguingly, inhibition of cysteine cathepsins or CTSB activity significantly increased the protein levels of PAI-1 (Figure 3A,B; Ca074Me: 250 ± 27%, *p* = 0.0007; E64d: 242 ± 58%, *p* = 0.013, *t*-test, *n* = 3). No significant changes in the protein levels of PLG or uPA were observed (not shown). We additionally confirmed these results in human TM with silenced expression of CTSB. Cells were transfected with siRNA targeting CTSB (siCTSB) or control siRNA (siNC) for 72 h and treated with TGFβ2 (10 ng/mL, 48 h). TGFβ2 is a known upregulator of PAI-1 and was used as a positive control. PAI-1 protein levels were evaluated by WB in whole cell lysates and conditioned media; mRNA PAI-1 levels were quantified by qPCR. As seen in Figure 3C–E, siCTSB knockdown significantly increased both intracellular and secreted protein levels of PAI-1. Higher PAI-1 mRNA levels were also observed in siCTSB-transfected cultures (Figure 3F).

### 3.4. CTSB Is Secreted into the Culture Media in Response to Cyclic Mechanical Stretch

**Figure 4:**
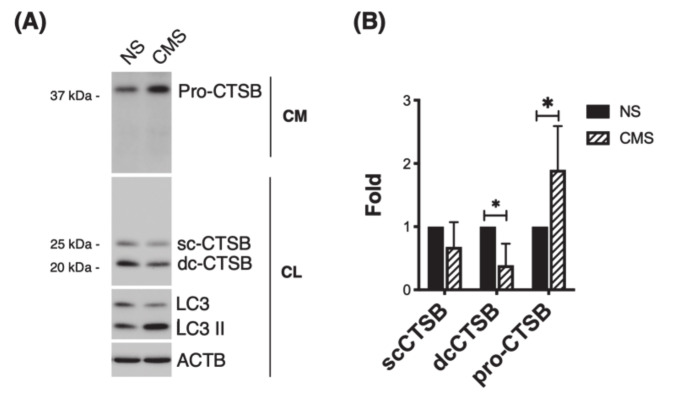
CTSB is secreted into the culture media in response to CMS. (**A**) Protein levels of CTSB in whole-cell lysates (5 μg) and culture media (15 μL) from hTM cells subjected to CMS (8% elongation, 24 h), compared to non-stretched controls. (**B**) Relative quantification of pro-CTSB, sc-CTSB, and dc-CTSB calculated from the densitometric analysis of the bands and compared to non-stretched controls. Data are shown as the mean ± S.D. * *p* < 0.05; two-tailed paired Student’s *t*-test, *n* = 4 and 5 for cell lysate and culture medium, respectively. NS: non-stretched; CMS: cyclic mechanical stretch; CM: culture media; CL: cell lysate. LC3 serves as control for mechanical stress.

Cells in the TM are exposed to mechanical stretch due to continuous changes in IOP. It is well-known that TM cells sense and respond to those mechanical forces by eliciting a number of responses, among them ECM remodeling, which are believed to be directed at maintaining IOP homeostasis [32,33]. We wanted to check whether mechanical stretch could affect expression or secretion of CTSB. For this, we subjected three primary cultures of human TM cells to cyclic mechanical stress (8% elongation, 24 h). CTSB expression was evaluated in whole cell lysates and culture media by WB. As seen in Figure 4A and quantified in 4B, a decrease in intracellular CTSB (single- and double-chain forms) was found in the stretched cultures (sc-CTSB: 0.68 ± 0.39 folds; dc-CTSB: 0.39 ± 0.34 folds, *p* < 0.05, *n* = 4). In contrast, higher levels of CTSB (pro-CTSB) were observed in mechanically stretched cells.

### 3.5. Downregulation of CTSB Decreases TGFβ1 and TGFβ2 and Modulates Smad2/3 Signaling Pathway

**Figure 5:**
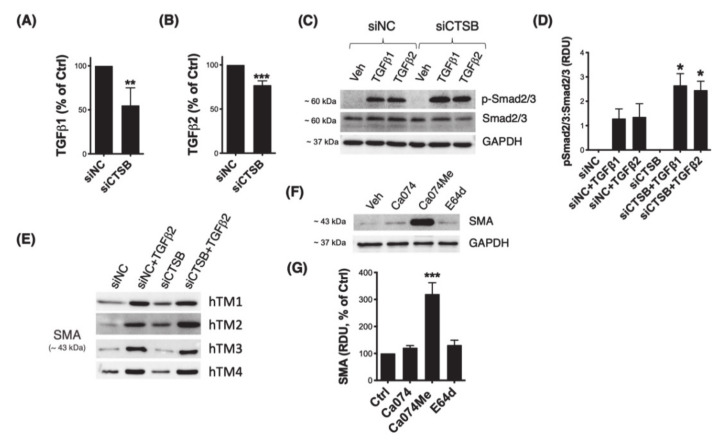
Effect of CTSB deficiency on TGFβ signaling. Secreted levels of (**A**) TGFβ1 and (**B**) TGFβ2 were quantified by ELISA in culture media from hTM primary cultures transfected with siCTSB or siNC control for 72 h. Data are shown as the mean ± S.D. **, *p* < 0.01; ***, *p* < 0.001, *t*-test, n_TGFβ1_ = 6, n_TGFβ2_ = 4. (**C**) Protein levels of pSmad2/3 and Smad2/3 in whole-cell lysates (5 μg) from hTM cells transfected with siCTSB or siNC for 72 h and subjected to TGFβ1 (10 ng/mL) or TGFβ2 (10 ng/mL) treatment for 48 h. Relative pSmad2/3 protein levels calculated from densitometric analysis of the bands are represented in (**D**). Data are shown as the mean ± S.D. *, *p* < 0.05 ANOVA with Tukey comparison tests, *n* = 3. (**E**) Protein levels of SMA in whole-cell lysates (5 μg) from four independent hTM strains transfected with siCTSB or siNC for 72 h and subjected to TGFβ2 (10 ng/mL) or TGFβ2 (10 ng/mL) treatment for 48 h. (**F**) Protein levels of SMA in whole-cell lysates (5 μg) from hTM cells treated with Ca074 (40 μM), Ca074Me (40 μM), or E64d (10 μM) for 24 h. Relative SMA protein levels calculated from densitometric analysis of the bands are represented in (**G**). Data are shown as the mean ± S.D. ***, *p* < 0.001, *t*-test, *n* = 4.

Recent reports in the literature have implicated CTSB in TGFβ signaling and epithelial–mesenchymal (EMT) transition [19,34,35]. Given the relevance of TGFβ and EMT in glaucoma pathogenesis, we investigated a potential regulatory effect of CTSB in these biological processes in TM cells. First, we quantified secreted levels of TGFβ1 and TGFβ2 in conditioned culture media from TM cells with silenced CTSB expression (siCTSB) via ELISA. Both TGFβ1 (Figure 5A, 55% ± 20%, *p* = 0.002, *t*-test, *n* = 6) and TGFβ2 (Figure 5B, 77% ± 5%, *p* < 0.0001, *t*-test, *n* = 4) were significantly decreased in TM cells with downregulated CTSB. Phosphorylation of Smad2/3 in response to TGFβ treatment was evaluated by WB. As seen in Figure 5C and quantified in 5D, cells with silenced CTSB expression displayed higher levels of p-Smad2/3 upon TGFβ1 (siCTSB: 2.656 ± 0.478 RDU vs. siNC: 1.292 ± 0.394 RDU, *p* < 0.05, *n* = 3) or TGFβ2 (siCTSB: 2.458 ± 0.367 RDU vs. siNC: 1.358 ± 0.545 RDU, *p* < 0.05, *n* = 3) treatments, compared to siNC-transfected cells. We additionally analyzed expression of the downstream TGFβ/Smad signaling myofibroblast marker, smooth muscle actin (SMA). Higher levels of SMA with CTSB knockdown were observed in two out of the four hTM strains evaluated (Figure 5E, Supplementary Materials Figure S1). Intriguingly, in all the cell strains tested, inhibition of CTSB activity by Ca074Me resulted in a marked increase in SMA (320 ± 42%, *p* < 0.0001, *t*-test, *n* = 4).

### 3.6. CTSB Knockout Mice Display Defective uPA Activation in the Iridocorneal Region

**Figure 6:**
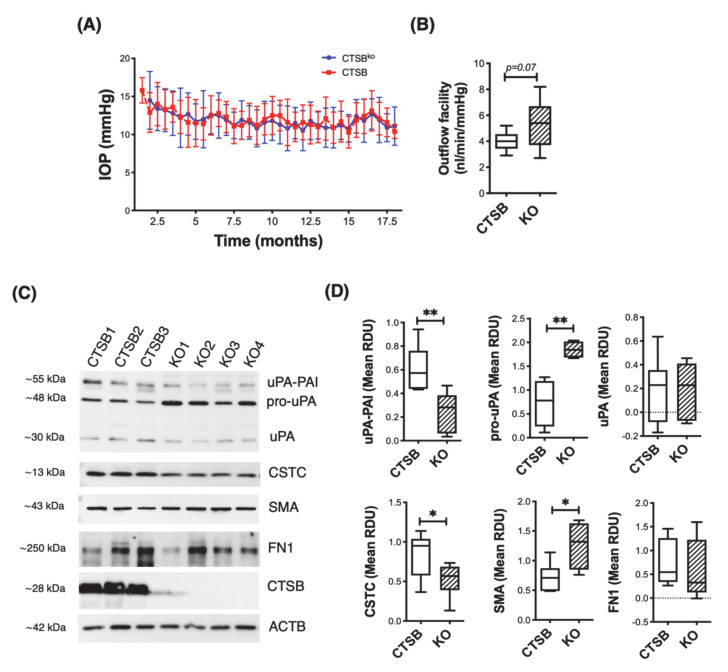
Effect of CTSB deficiency in outflow pathway physiology and morphology. (**A**) Mean IOP (mm Hg) over time in CTSB^ko^ and CTSB mice. Data are shown as the mean ± S.D., *n* = 20. (**B**) Mean outflow facility in CTSB^ko^ and CTSB mice (4 m.o.) evaluated ex-vivo with the iPerfusion system. Data are shown as the mean ± S.D., n_CTSB_ = 10, n_CTSBko_ = 11. (**C**) Protein levels of uPA (uPA/PAI, pro-uPA, uPA), CTSC, SMA, FN1, and CSTB evaluated by WB in whole cell lysates (5 μg) from primary hTM cells. Relative protein levels were calculated from densitometric analysis of the bands and represented in (**D**). Data are shown as the mean ± S.D. * *p* < 0.05, ** *p* < 0.01; *t*-test, *n* = 4–7.

Next, we investigated the potential effects of CTSB deficiency in outflow pathway tissue physiology. For this, we monitored IOP and outflow facility in CTSB deficient mice (CSTB^ko^) and littermate CTSB mice. As seen in Figure 6A, no difference in IOP was observed between CTSB and CTSB^ko^ mice, evaluated up to 18-month-old age. Interestingly, an increased in outflow facility was observed in CTSB^ko^ mice compared to CTSB control, although it did not reach statistical significance (Figure 6B, CTSB^ko^: 5.155 ± 1.836 nl/min/mmHg vs. CTSB: 4.03 ± 0.739 nL/min/mmHg, *p* = 0.07, *t*-test, *n* = 10). Based on the findings in our in vitro studies, we analyzed the expression levels of several components of the proteolytic cascade in dissected iridocorneal region tissues of CTSB^ko^ and littermates CTSB, based on antibody availability (Figure 6C, D). Absence of CSTB in the iridocorneal region tissue in CTSB^ko^ mice was confirmed. Compared to CTSB, CTSB^ko^ mice displayed a very significant increase in pro-uPA (CTSB^ko^: 1.850 ± 0.178 RDU vs. CTSB: 0.735 ± 0.494 RDU; *p* = 0.005, *t*-test, *n* = 4). This was accompanied by a decrease in uPA-PAI-1 levels (CTSB^ko^: 0.248 ± 0.167 RDU vs. CTSB: 0.610 ± 0.198 RDU; *p* = 0.006, *t*-test, *n* = 6). Decreased levels of cystatin C (CSTC), an endogenous CTSB inhibitor, were also found in KO mice (CTSB^ko^: 0.525 ± 0.215 RDU vs. CTSB: 0.838 ± 0.279 RDU; *p* = 0.04, *t*-test, n_CTSB_ = 7, n_CTSBko_ = 6). Additionally, CTSB^ko^ mice displayed higher levels of the fibrotic marker SMA (CTSB^ko^: 1.255 ± 0.398 RDU vs. CTSB: 0.738 ± 0.229 RDU; *p* = 0.016, *t*-test, n_CTSB_ = 7, n_CTSBko_ = 6), but no significant changes in fibronectin (FN1) were detected.

**Figure 7:**
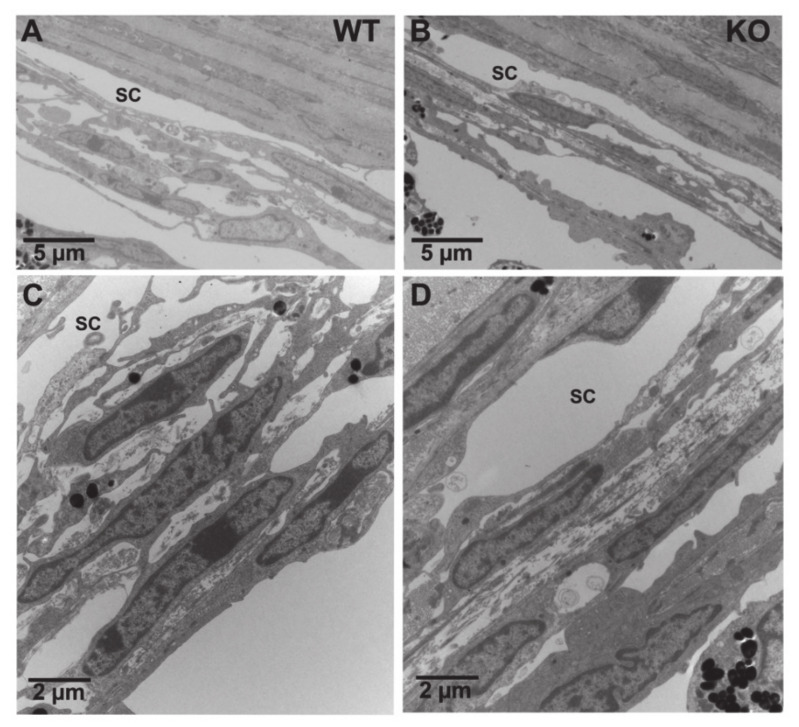
Morphological Comparison of Trabecular Meshwork between CTSB and CTSB^ko^ mouse eyes. (**A**–**C**): Representative electron microscopy images of the TM from CTSB mouse eyes at lower (**A**) and higher (**C**) magnifications. (**B**–**D**): Representative electron microscopy images of the TM from CTSB^ko^ mouse eyes at lower (**B**) and higher (**D**) magnifications. Compared to CTSB mouse eyes, no obvious differences were observed in the endothelial cell of Schlemm’s canal (SC), cells and extracellular matrix in the juxtacanalicular connective tissue regions, and the endothelial cells and extracellular matrix core of trabecular beams in CTSB^ko^ mouse eyes.

EM morphometric analysis showed no gross changes in outflow pathway morphology in CTSB^ko^ mice (Figure 7). Both cells and ECM look normal in the TM of KO mice compared to CTSB mouse eyes; no obvious differences were observed in either the endothelial cell of Schlemm’s canal, juxtacanalicular connective tissue regions, or TM endothelial lining cells between the two groups.

## 4. Discussion

In this manuscript, we report for the first time that CTSB localizes in the caveolae and participates in the pericellular degradation of ECM in TM cells. We also report here a novel role of CTSB in regulating the expression of PAI-1 and TGFβ/Smad signaling in vitro and in vivo in CTSB^ko^ mice.

Caveolae are unique invaginated lipid-rich regions of the plasma membrane highly abundant in mechanically stretched cells that play a major role in regulating cell signaling, lipid homeostasis, and adaptation to membrane tension. Although less investigated, emerging studies are showing that caveolae are important regulators of ECM remodeling, acting as focal proteolytic degradation sites. Disruption of caveolae formation in Cav1-deficient cells results in abnormal expression of matrix proteins [36,37,38]. In TM cells, Cav1 deficiency has been shown to alter the physiological catabolism of ECM and modulate outflow resistance [39]. First, many proteases implicated in ECM remodeling are associated or localized to the caveolae. Second, caveolae mediates internalization, via endocytosis, of partially degraded ECM fragments and targets them for final degradation within lysosomes. Along these lines, our studies revealed the localization of members of the proteolytic cascade, pro-uPA and uPAR, along with that of the cysteine cathepsin CTSB in Cav-1-enriched fractions from TM cells. Interestingly, AnxA2, a reported cell surface receptor for tPA and CTSB was also localized in the caveolae [28]. CTSB in the caveolae was found in its inactive (pro-CTSB) and as a single chain (sc-CTSB) forms, consistent with the notion that sc- to dc- maturation occurs in lysosomes. An advantage of localization of CTSB in the caveolae is the quick availability. Our results showed increased secretion of pro-CTSB in response of mechanical stretch. It is plausible that mechanical deformation of caveolae with mechanical forces triggers the release of the pro-enzyme and be one of the contributing factors initiating ECM remodeling in stretched cells.

Using a live-cell proteolysis assay in combination with CTSB inhibitors, we confirmed the contribution of cell surface CTSB in the pericellular degradation of ECM components. ECM degradation at the cell surface occurs by the coordinated action of different types of proteases and their receptors. In a proposed model for tumor cells, activation of receptor-bound-pro-CTSB (AnxA2/CTSB) at the caveolae triggers the uPA/plasminogen/plasmin proteolytic cascade that culminates with MMP activation and ECM breakdown. CTSB can also directly activate some MMPs. Very interestingly, pharmacological inhibition or silencing of CTSB resulted in transcriptional upregulation and increased secretion of PAI-1 in TM cells. PAI-1 is a member of the superfamily of serine-protease inhibitors or serpins and potent inhibitor or tPA and uPA. PAI-1 binds to uPA/uPAR complex and targets uPAR for degradation. To our knowledge, this is the first time that regulation of PAI-1 expression by CTSB is reported and adds a novel mechanism by which CTSB can modulate ECM proteolysis in addition to proteolytic activation. Consistent with these results, we observed increased levels of pro-uPA in the dissected iridocorneal mouse tissue of CTSB^ko^ mice compared to CTSB controls. CTSB^ko^ mice also displayed lower levels of CTSC, an endogenous inhibitor for CTSB, suggesting a regulatory feedback between the two proteins.

Noteworthy are the increased levels of SMA in the iridocorneal region of CTSB^ko^ mice. A marked increase in SMA protein levels was also identified in vitro in hTM cells treated with the CTSB inhibitor Ca074Me. One could argue that the observed increase in pSmad2/3 levels could explain the increase in SMA. However, silencing CTSB expression elevated SMA levels in just two out of the four cell strains tested, while elevated pSmad2/3 was observed in all of them. Intriguingly, the capability of CTSB to modulate SMA seems to be a cell strain-intrinsic factor, since independently conducted replicate experiments using the same cell strains yield the same outcomes. It is plausible that elevated SMA levels might also result from defective CTSB-mediated degradation of either SM or SMA negative transactivation signal. That might partially explain the why irreversible inactivation of CTSB activity with Ca074Me resulted in such remarkable increase in SMA protein levels. This, however, requires further investigation.

A role of CTSB in EMT and tissue fibrosis has been investigated in a very recent work [34]. In this elegant study, Zhang et al. report a differential role of cysteine cathepsins in TGFβ signaling and kidney fibrosis. Interestingly, using a model of unilateral ureteral obstruction, the authors found that CTSB or CTSL deficiency in mice exacerbates kidney fibrosis, whereas CTSS or CTSK deficiency protects it. Similar to our own results, they detected higher expression levels of SMA in CTSB^ko^ mice. More, also as in siCTSB-transfected TM cells, tubular epithelial cells deficient in CTSB showed increased pSmad2/3 in response to TGFβ treatment. Intriguingly, they found that this resulted from the interaction of membrane surface CTSB with TGFBR1, targeting TGFBR1 for degradation and abolishing downstream TGFβ signaling. CTSB has been also shown to regulate TGFβ pathway by participating in the intracellular proteolytic activation and maturation of TGFβ1 [35]. Although not confirmed in TM cells, this could certainly explain the observed lower secreted levels of TGFβ1 and TGFβ2 in siCTSB-transfected TM cells.

Fibrosis and EMT transition have been linked to the pathogenesis of glaucoma. The fact that despite the increase in SMA and diminished pro-UPA activation CTSB^ko^ mice showed a trend in higher—than rather lower—outflow facility and no significant morphological or physiological changes in the outflow pathway tissues was somehow a little disappointing and unexpected. As we previously reported, glaucomatous TM cells display lower CTSB protein levels [40]. Several contributing factors can explain these results. First and foremost is the known compensatory role of cysteines cathepsins, in particular between CTSB and CTSL [41]. The second factor is age. While no differences in IOP were noted over time, outflow facilities and morphological analysis were conducted just in young mice. The third factor is TGFβ. As indicated by our results presented here and those in [34] [35], there is a complex relationship between CTSB and TGFβ signaling. While decreased levels of TGFβ1 and TGFβ2 were noted in TM cells with knockdown expression of CTSB, the concentration of TGFβ2 in AH from glaucomatous eyes is known to be higher than that in age-matched control eyes. It is therefore expected that effects of CTSB differ in a physiological compared to a pro-fibrotic environment. Future experiments will be directed at exploring the effect of aging and pro-fibrotic environment in CTSB-mediated activation of the proteolytic cascade and ECM remodeling.

## 5. Conclusions

In summary, we have identified here CTSB as a novel mechanosensitive player regulator of the proteolytic cascade and TGFβ signaling in TM cells. Small drugs targeting cathepsins are currently being developed and tested in clinical trials to treat diseases with imbalance ECM turnover. Similar approaches targeted at enhancing CTSB activity or restoring CTSB levels in the glaucomatous TM could be developed to attenuate fibrosis and ECM deposition in the TM in glaucoma.

## Figures and Tables

**Table 1:** List of antibodies and dilutions.

Protein	Company	Catalog	Dilution
CTSB	Cell Signaling	31718S	1:1000
Cav1	Sigma	C3237	1:1000
Annexin	Santa Cruz Biotechnology	Sc-28385	1:1000
uPA	R&D	MAB9185	1:1000
uPAR	R&D	MAB807	1:1000
PAI-1	Santa Cruz Biotechnology	sc-5297	1:250
GAPDH	Santa Cruz Biotechnology	sc-47724	1:500
CSTC	Millipore	ABC20	1:1000
SMA	Sigma	A2547	1:1000
FN1	Santa Cruz Biotechnology	31718S	1:2000
ACTB	Santa Cruz Biotechnology	Sc-69879	1:500

## Supplementary Materials

The following are available online at https://www.mdpi.com/2077-0383/10/1/78/s1, Figure S1: Effect of CTSB deficiency on SMA. Protein levels of SMA in whole-cell lysates (5 μg) from four independent hTM strains transfected with siCTSB or siNC for 72 h and subjected to TGFβ2 (10 ng/mL) or TGFβ2 (10 ng/mL) treatment for 48 h. Graph represents relative protein levels calculated from densitometric analysis of the bands.
