# Supplementary figures and images for: Cathepsin B Localizes in the Caveolae and Participates in the Proteolytic Cascade in Trabecular Meshwork Cells. Potential New Drug Target for the Treatment of Glaucoma
Source: J Clin Med. 2020 Dec 28;10(1):78. doi: 10.3390/jcm10010078 (PMC7795952; doi:10.3390/jcm10010078)

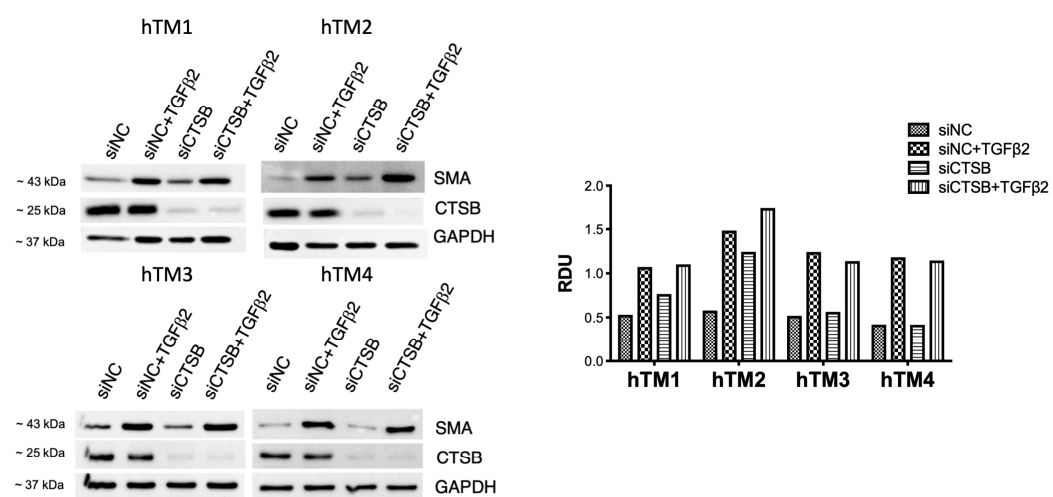

Figure S1 Please add the title and other figure footer for figure S1

Supplement: Supplementary file 1 [file jcm-10-00078-s001.pdf]
